# Factors Associated With “Sterile Cystic Formation” Following the Application of BioGlue® in Brain Surgery and Review of Literature

**DOI:** 10.7759/cureus.75523

**Published:** 2024-12-11

**Authors:** Methee Wongsirisuwan, Tatre Jantarakolica, Nichapa Lerthirunvibul

**Affiliations:** 1 Neurosurgery, Rangsit University, Bangkok, THA; 2 Neurosurgery, Rajavithi Hospital, Bangkok, THA; 3 Economics, Thammasat University, Bangkok, THA; 4 Emergency Medicine, King Chulalongkorn Memorial Hospital, Bangkok, THA

**Keywords:** bioglue®, brain surgery, infection, inflammation, wound dehiscence

## Abstract

Introduction

BioGlue® (CryoLife, Inc, Kennesaw, GA), despite being claimed to be a safe and harmless sealant, reportedly has several adverse effects including surgical wound dehiscence. This study aimed to examine the factors that may contribute to this unfavorable outcome in cranial surgery.

Methods

A retrospective cross-sectional analysis was conducted on patients who underwent brain surgery with the use of BioGlue® between January 2015 and December 2022. One hundred and two individuals were enrolled and classified into two categories based on the incidence of surgical wound complications. The patients’ demography, operative details, and the nature of their surgical wound problems were evaluated.

Results

Out of 102 individuals, 15 experienced postoperative wound complications. Fluid resembling pus was noted; however, laboratory cultures yielded negative results. Complications were significantly more prevalent (seven times higher) among individuals utilizing BioGlue® in proximity to titanium plates for skull defect correction. Patients using the 5 mL pre-filled BioGlue® device had a 2.8 times higher likelihood of complications than the 2 mL version. Using the patient's cranial bone as a barrier during surgery reduced the risk of complications.

Conclusions

Neurosurgeons should note possible adverse reactions of using a sealant for cranial surgery that include inflammation and wound separation. Treatment should include thorough cleansing and complete removal of BioGlue®. Two prevalent risk factors were the close proximity (direct contact) of BioGlue® to the adjacent titanium plate used for covering skull defect and the volume of sealant used (5 mL or 2 mL).

## Introduction

BioGlue® (CryoLife, Inc, Kennesaw, GA), a medical adhesive composed of glutaraldehyde and bovine serum albumin, has revolutionized the field of neurosurgery. One of the primary benefits of BioGlue® is its ability to create a durable and enduring closure within a minute, thereby preventing the escape of cerebrospinal fluid (CSF) from the surgical area through dural defects which is essential for lowering the possibility of complications such as infection, intracranial hypotension, and pseudomeningocele. These post-operative complications can negatively impact a patient's recovery and overall success of the surgical procedure. The properties of BioGlue® enable it to adhere to various surfaces including challenging anatomical sites which provides neurosurgeons with a reliable and efficient means of tissue sealing. However, neurosurgeons must be aware of potential adverse effects and complications associated with the utilization of this adhesive, particularly in certain patient populations. Numerous global medical studies have acknowledged these adverse outcomes, yet none has elucidated the relevant factors contributing to these unfavorable events [1-5].

Based on the author's extensive surgical experience and familiarity with various materials used to prevent CSF leakage including Tisseel fibrin sealant, Duragen, Hemopatch, and BioGlue®, it has been observed that BioGlue® is the most effective material in preventing CSF leakage. Nevertheless, certain individuals have had surgical wound problems after the application of BioGlue®, which were absent in cases where alternative sealant materials were employed. With various reports on the adverse effects of BioGlue®, this study aimed to investigate the potential factors that may increase the likelihood of postoperative wound complications related to the utilization of BioGlue® following brain surgeries in healthy patients. If neurosurgeons are made aware of these potential risk factors, the likelihood of wound complications when using BioGlue® may be reduced.

## Materials and methods

This retrospective observational and monocentric cross-sectional study collected medical records of patients who underwent brain operations using BioGlue® as a sealant from January 1, 2015 to December 31, 2022. All medical information was collected from the hospital’s electronic medical record. Information collected included patient’s demographic data, diagnosis, and preoperative, operative, and postoperative outcomes relating to the surgical wound that may be associated with the use of BioGlue®. A follow-up period of at least one year was implemented in all cases. In all cases, the operations were performed by a single neurosurgeon who has more than 20 years of experience in brain surgery.

Patients were included if they had undergone brain surgery and received BioGlue® as a sealant to prevent CSF leakage as part of their surgical procedure. In all cases, BioGlue® was stored, handled, and prepared according to the manufacturer's recommendations to ensure its sterility and effectiveness. Exclusion criteria were patients lost to follow-up or incomplete medical records; patients with history of Bovine substance hypersensitivity, patients with medical history of radiotherapy in the area of the surgical field; patients with a medical history of previous surgery performed through the same surgical incision; patients with comorbidities significantly impacting the immune system, such as uncontrolled diabetes mellitus, receiving corticosteroids or immunosuppressant drugs, or having autoimmune diseases; and patients with a history of systemic infection/sepsis within the three months preceding brain surgery.

The study hypothesized that the chemical composition of BioGlue (either glutaraldehyde or bovine albumin) increases the risk of surgical wound complications. The primary aim was to analyze patient characteristics, surgical variables, and postoperative wound outcomes such as surgical site inflammation, wound dehiscence, seroma, delayed wound healing, and the onset of problems following the application of BioGlue® in the surgical field. The secondary aim was to evaluate the possible relevant factors that may increase the likelihood of surgical wound problems after the use of BioGlue® as a sealant.

**Figure 1:**
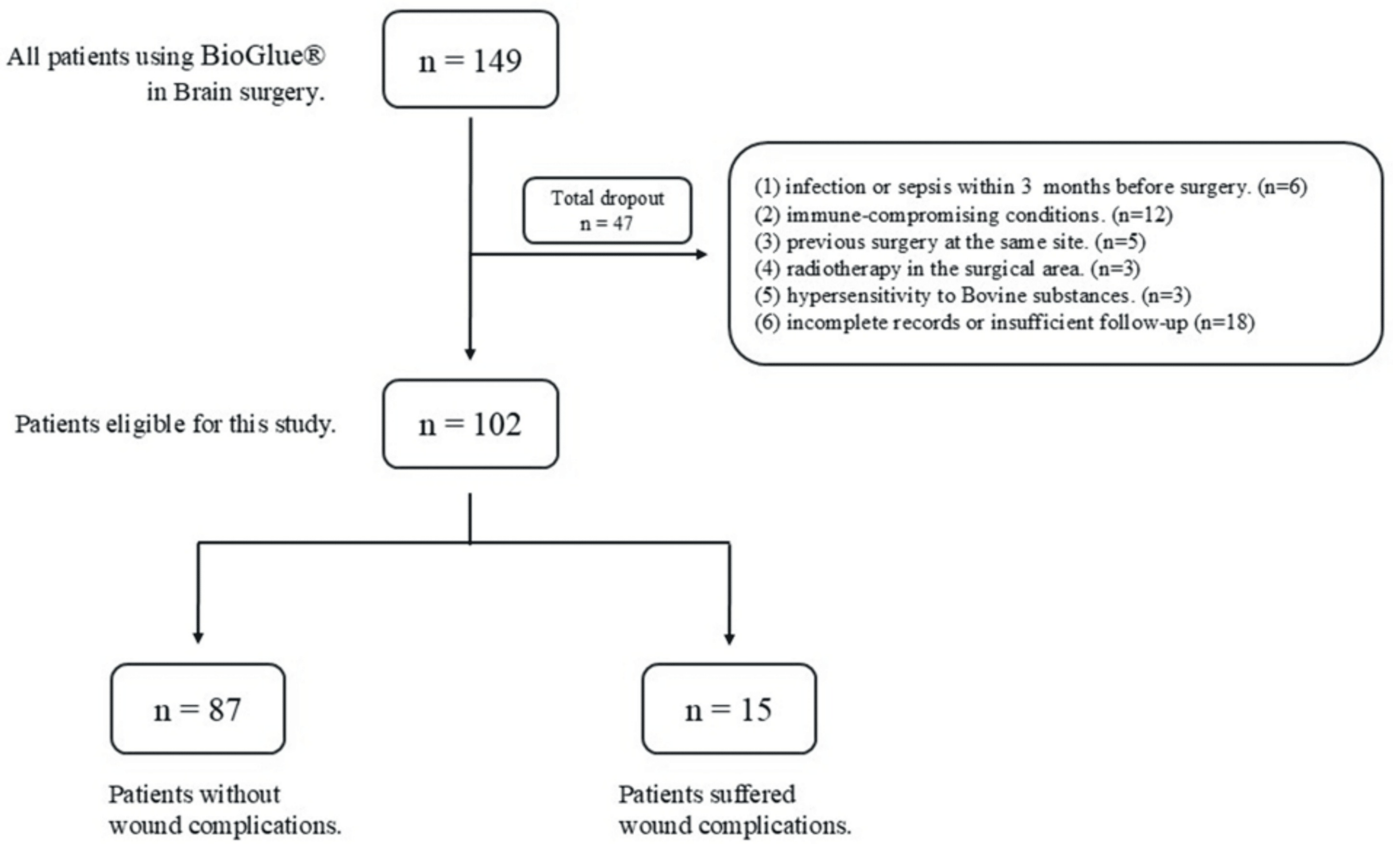
Flow diagram of the eligibility.

There was a total of 149 cases received BioGlue® as a sealant to prevent CSF leakage after brain surgery. However, 47 cases were excluded, and 102 individuals were eligible and included in the study (Figure 1).

The patients underwent either craniectomy or craniotomy surgery and cranioplasty was done using either autologous cranial bones or titanium plates. Two different volumes of BioGlue® used during the intracranial surgery: 2 mL and 5 mL pre-filled syringes. The patients' ages were categorized into two groups (under 60 years and 60 years and above) based on the onset of immunosenescence, which typically begins in the sixth decade of life [6].

Collected data included a comprehensive review of medical records, operative notes, and postoperative follow-up documents. Patient demographics, age, gender, and pre-existing medical problems were recorded. The research collected various surgical parameters including the specific type of brain surgery conducted, the volume of pre-filled BioGlue® syringes utilized (either 2 mL or 5 mL), and the placement of a titanium plate that is in direct contact with BioGlue® for a cranial defect post-craniectomy. The low-profile titanium mesh plates utilized in all cases requiring coverage of skull defects are manufactured by Jeil Medical Corporation (Seoul, Republic of Korea) with measurements of 0.3 millimeters (mm) in thickness, whereas the self-drilling titanium screw has a diameter of 0.14 mm.

Statistical analysis

Statistical analysis using STATA 17.0 (StataCorp., College Station, TX) was conducted to determine the associations between relevant factors and surgical wound problems. Categorical variables were analyzed using Chi-Square and Fisher's exact tests, and logistic regression analysis was performed to identify independent risk factors for surgical wound complications, with adjustments for potential confounders. Gender and age of patients were considered as control variables in the analysis, and multivariate analysis using a logistic regression model was applied to determine factors influencing wound complications. The model was estimated using the maximum likelihood estimation method.

## Results

The total number of consecutive cases of receiving BioGlue® as a sealant to prevent CSF leakage after brain surgery was 149. Following applying the exclusion criteria, 47 cases were dropped out, resulting in 102 individuals who satisfied the criteria specified; 15 (14.7%) had suffered postoperative wound complications. The mean ages of the patients without and with wound complications were 55.56 ± 18.38 and 57.00 ± 22.70 years, respectively. The surgical site complications appeared at a mean duration of 22.07 ± 19.8 days following the surgical procedure. The average follow-up period was at least 14.08 ± 1.2 months. The nature of the wound complications was similar in all cases. The majority of patients presented with symptoms of swelling and wound dehiscence at the sutured surgical incision site with turbid white or yellowish exudate. Some wounds achieved spontaneous healing, while others experienced further dehiscence and increased discharge production. Both aerobic and anaerobic cultures were performed, but no evidence of bacterial infection was found. No patient complained of pain or tenderness at the surgical incision site and exhibited no signs of fever which are typical indicators of surgical site infection.

**Figure 2:**
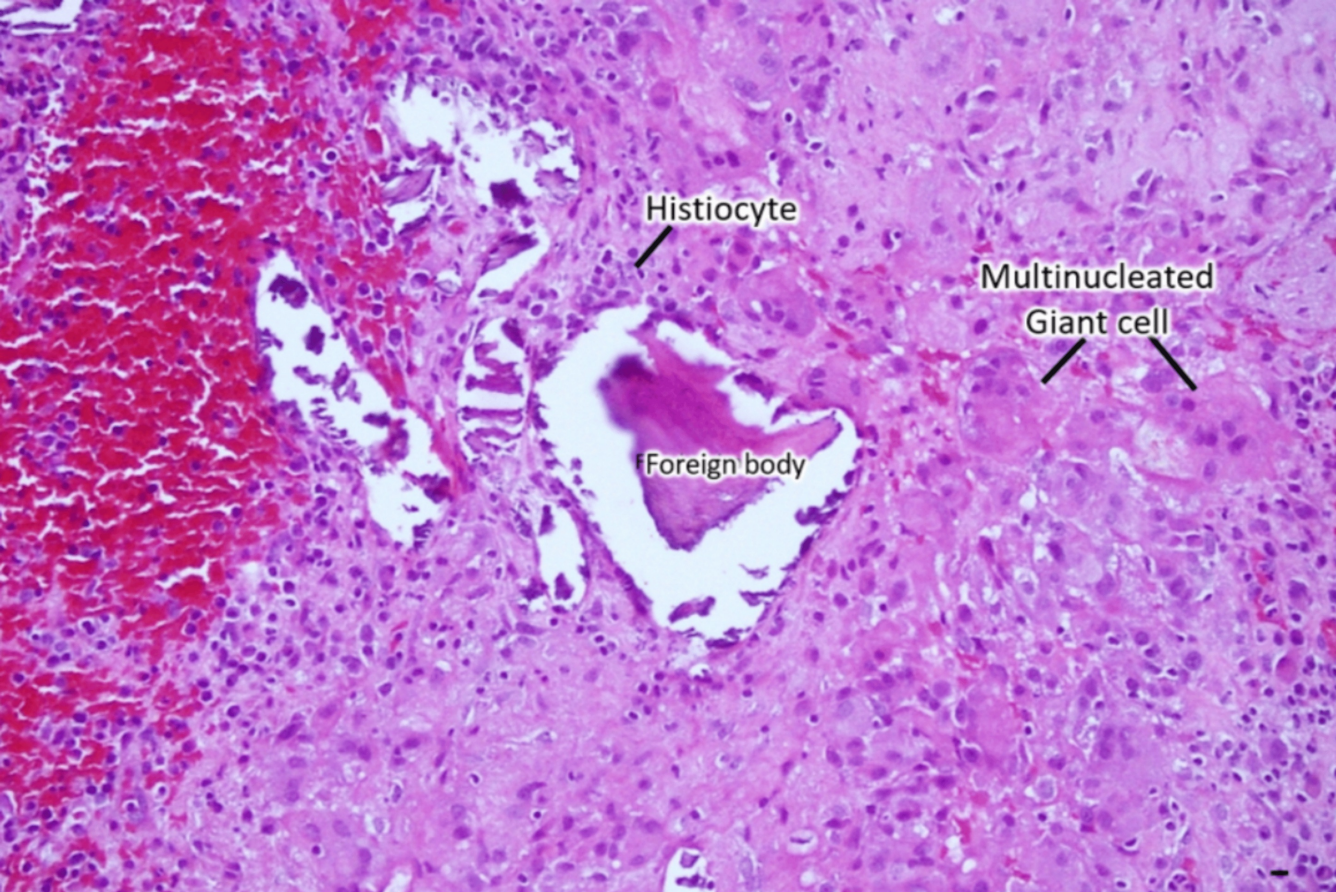
Histocytes and multinucleated giant cells surrounding the foreign body (BioGlue®) without any microorganisms. H&E stain, Magnification: 300x, Scale bar: 10 µm.

**Figure 3:**
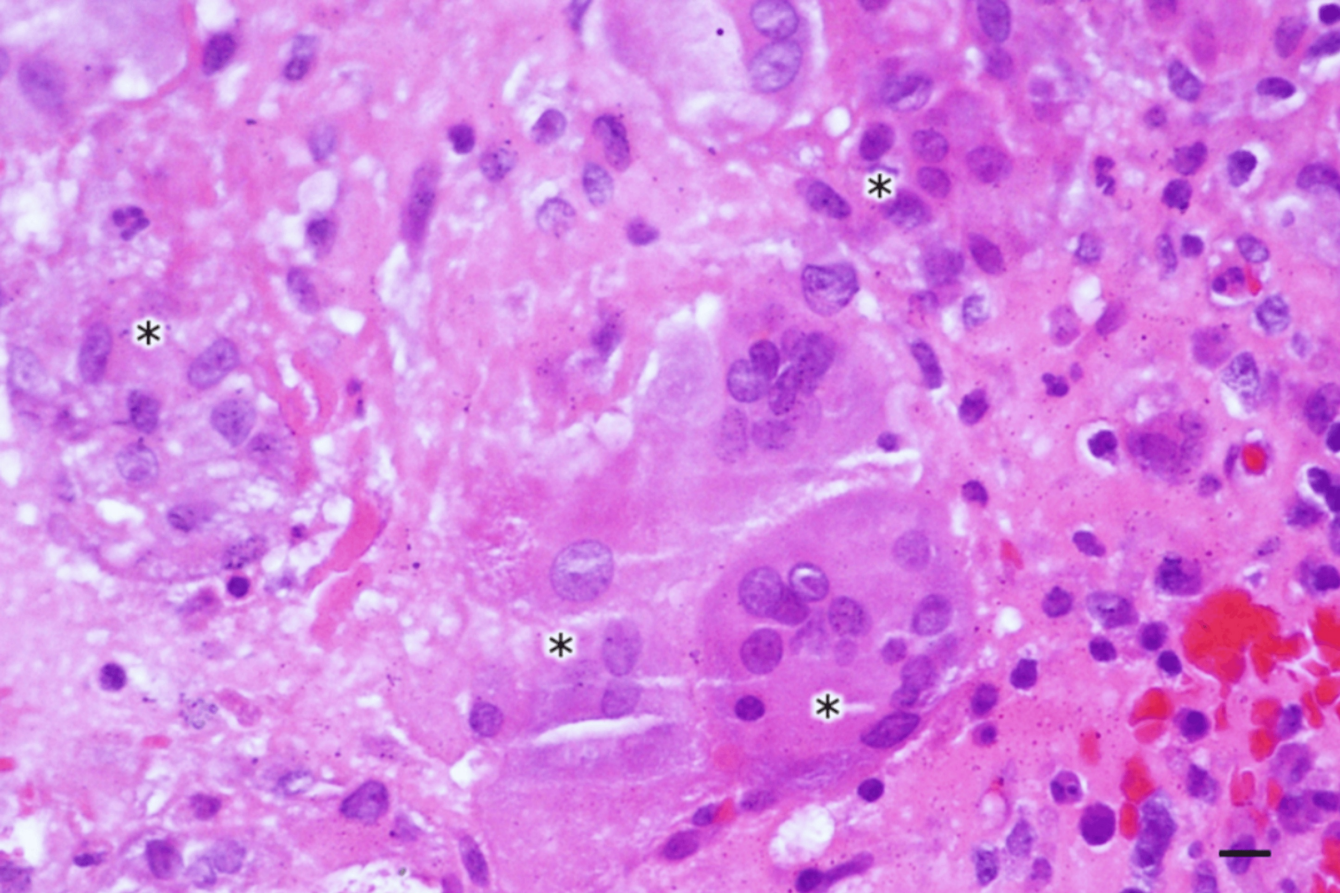
Granuloma formation process. * - multinucleated giant cells coalesce to form granuloma. H&E stain, Magnification: 500x, Scale bar: 10 µm.

**Figure 4:**
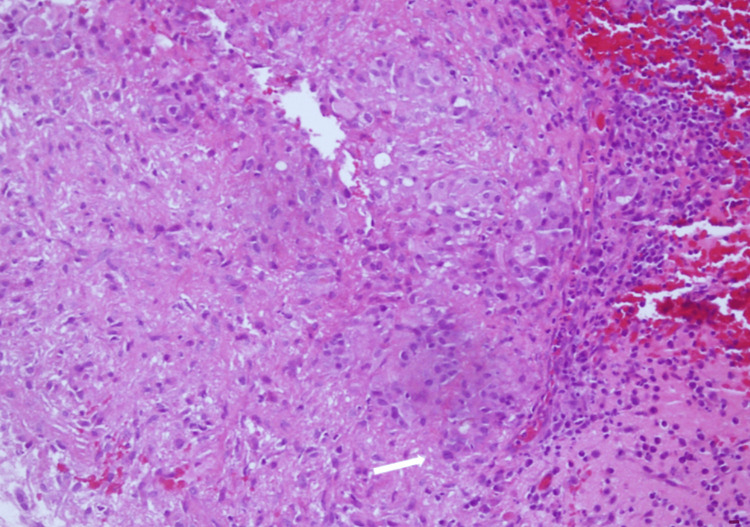
Foreign body granuloma: lots of multinucleated giant cells (white arrow). H&E stain, Magnification: 300x, Scale bar: 10 µm.

**Figure 5:**
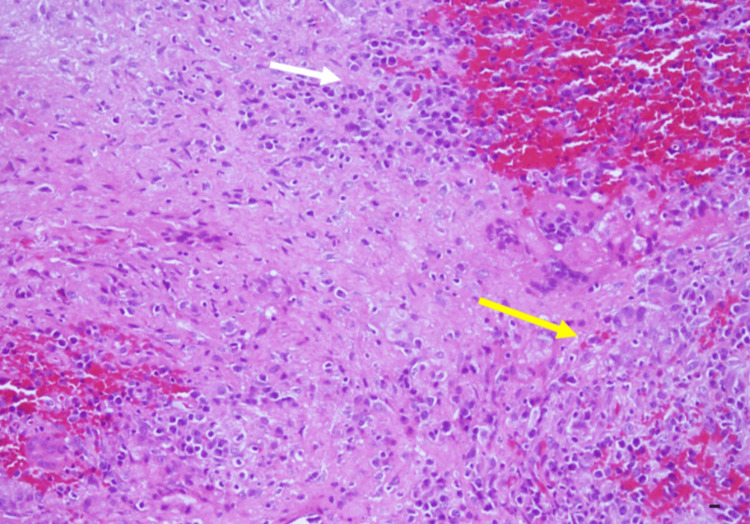
Inflammatory cells and histocytes with extravasation of blood (white arrow), and multinucleated giant cells (yellow arrow). H&E stain, Magnification: 300x, Scale bar: 10 µm.

Incision and drainage (I&D) therapy initially alleviated the wound problems; however, swelling recurred in most cases even after I&D. Eventually, wound irrigation was necessitated to ensure thorough cleansing and elimination of residual BioGlue® adhesive and removal of titanium metal plates that served as cranial coverings. The wounds subsequently healed completely without the need for any antimicrobial medication. Histopathological analysis of the subcutaneous tissue and the discharge confirmed “sterile cystic formation” without any infection. The results revealed the presence of chronic inflammation evidenced by multinucleated giant cells and histiocytes (Figure 2), granuloma formation process (Figures 3, 4), and inflammatory cells with histiocytes (Figure 5).

Table 1 presents an overview of the patient’s clinical data and the corresponding statistical findings comparing the two groups: patients with and without wound complications following the application of BioGlue®. Of 102 patients, 87 and 15 cases received craniectomy and craniotomy surgeries, respectively. Titanium was utilized in 47 cases, whereas the other 55 patients did not have titanium plates. The 2 and 5 mL of pre-filled BioGlue® syringes were used in 83 and 19 cases, respectively. There was an equal ratio of male to female patients included in the study with 51 males and 51 females. There were 59 and 43 participants with ages below and above 60, respectively.

**Table 1:** Overview of the patient’s clinical data and the corresponding statistical findings of the two groups. Note: * Significant at 10%, ** Significant at 5%, and *** Significant at 1%.

	Complication		Total	Pearson chi-square	P-value	Fisher’s exact test (p-value)
Total	87	15	102			
	(85.3%)	(14.7%)	(100%)			
Operation				0.0264	0.8710	0.9999
Craniectomy	74	13	87			
	(85.1%)	(14.9%)	(100%)			
Craniotomy	13	2	15			
	(86.7%)	(13.3%)	(100%)			
Utilization of Titanium				8.1445	0.0040 ***	0.0050 ***
No	52	3	55			
	(94.5%)		(100%)			
Yes	35	12	47			
	(74.5%)	(25.5%)	(100%)			
BioGlue				2.5091	0.1130	0.1480
2 mL	73	10	83			
	(88%)	(12.0%)	(100%)			
5 mL	14	5	19			
	(73.7%)	(26.3%)	(100%)			
Gender				0.0782	0.7800	0.9999
Male	44	7	51			
	(86.3%)	(13.7%)	(100%)			
Female	43	8	51			
	(84.3%)	(15.7%)	(100%)			
Age				0.1467	0.7020	0.7800
Below 60	51	8	59			
			(100%)			
60 and above	36	7	43			
	(83.7%)	(16.3%)	(100%)			

In patients without wound complications, 74 and 13 cases underwent craniectomy and craniotomy surgeries, respectively. In cases with post-operative suture site complications, 13 and two patients received craniectomy and craniotomy operations, respectively. The results of the Pearson Chi-square test, p-value, and Fisher’s exact test for comparing the occurrence of wound complications between craniectomy and craniotomy were 0.0264, 0.8710, and 0.9999, respectively. In the 47 patients whose cranial defect was repaired using titanium, 12 cases developed post-operative wound complications. The Pearson Chi-square test, p-value, and Fisher’s exact test for utilization of titanium were 8.1445 significant at 1%, 0.0040, and 0.0050, respectively.

Of the 83 patients who received 2 mL prefilled BioGlue® syringes, 10 cases experienced wound complications. While five of 19 patients who received 5 mL BioGlue® syringes had post-operative suture site complications. Comparing the use of 2 and 5 mL prefilled BioGlue® syringes, the Pearson Chi-square test, p-value, and Fisher’s exact test yielded 2.5091, 0.1130, and 0.1480, respectively. A number of seven and eight male and female patients, respectively, had wound complications from a total of 51 patients in each gender group with Pearson Chi-square test, p-value, and Fisher’s exact test of 0.0782, 0.7800, and 0.9999. Wound complications were found in eight and seven patients below and above the age of 60, respectively. Whereas 51 and 36 patients below and above 60 years of age, respectively, did not encounter suture wound issues. The Pearson Chi-square test, p-value, and Fisher’s exact test for comparison between incidences of wound complications in the two age groups were 0.1467, 0.7020, and 0.7800, respectively.

**Figure 6:**
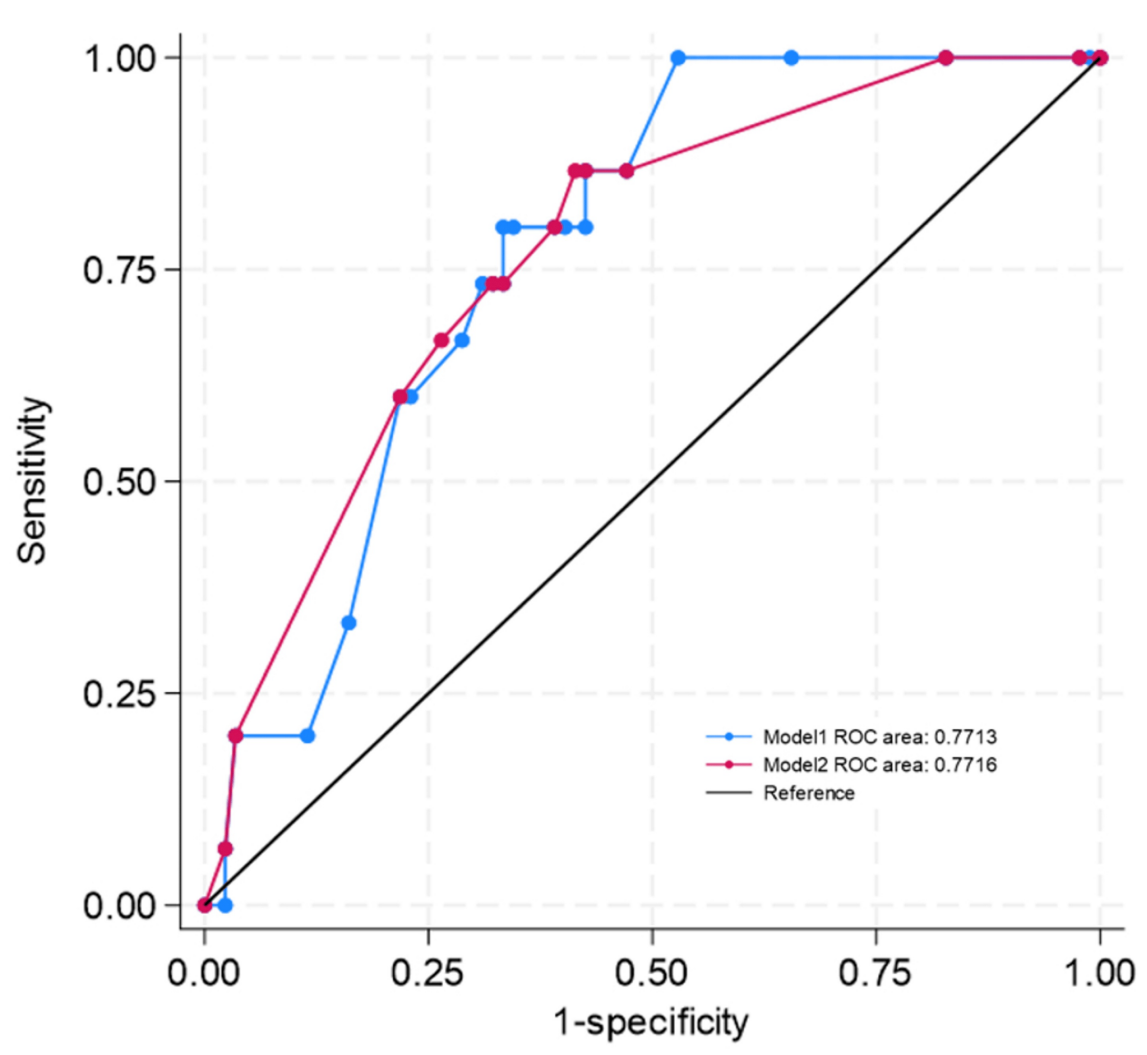
ROC comparison of ROC curves based on the estimated results of Model 1 and those of Model 2 in Table 2. ROC - receiver-operating characteristic

In a cohort of patients experiencing wound complications following the use of BioGlue®, we identified three potential factors that may exhibit significant associations with the development of surgical wound inflammation. These factors include the utilization of titanium, the closure of the skull defect, and the size of the pre-filled BioGlue®. We also included the gender and age of patients as control variables in the analysis. Therefore, this study employed multivariate analysis with a logistic regression model to identify the factors influencing the chance of wound complications. We estimated the model using the maximum likelihood estimation method. As shown in Table 2, all statistical indices illustrate that the estimated results of logistic regression models can statistically significantly explain the probability of occurrence of a wound complication. Model 2 produces superior results, indicating that the patient's gender and age do not solely determine the possibility of wound complications, but rather the interaction between these two factors. Males under the age of 60 are less likely to experience wound complications. Patients who undergo cranioplasty utilizing a titanium plate exhibit a greater chance of encountering surgical wound complications, with an odds ratio of 7.265. Patients who use a 5 mL pre-filled BioGlue® are more likely to encounter complications than individuals who employ the 2 mL version, with odds of up to 2.8 times greater. However, if the cranial defect were repaired using autologous cranial bone, which serves as a barrier between the BioGlue® and the surrounding soft tissue or titanium plate, the likelihood of encountering the issues would be reduced, lowering the odd ratio by 0.3 times compared to the ratio of the group that did not undergo cranioplasty. These observations derived from the statistical analysis are consistent with clinical experience. Another observation reveals that males under 60 have a slightly reduced odd ratio by half of experiencing wound-related issues. Details of the statistical analysis are shown in Table 2. Figure 6 illustrates the comparison of receiver-operating characteristic (ROC) curves based on the estimated results of Model 1 and those of Model 2. The results indicate that Model 2 provides better results in terms of predicting power (wound complication).

**Table 2:** The potential factors related to surgical wound complications following the use of BioGlue® based on the estimated results of logistic regression models. Note: Values in parentheses (.) are standard errors of the odds ratio (OR). Values in square brackets [.] represent 95% confidence interval (CI) from lower to upper bound of the OR. * Significant at 10%, ** Significant at 5%, and *** Significant at 1%. AUC = Area Under ROC Curve.

	Model 1	Model 2
	Odds ratio	Odds ratio
Titanium	6.901 ***	7.265 ***
	(0.322)	(0.312)
	[6.84, 6.96]	[6.88, 7.90]
Craniotomy	0.300 ***	0.299 ***
	(0.031)	(0.044)
	[0.25, 0.37]	[0.22, 0.40]
BioGlue® 5 mL	2.706 ***	2.821 ***
	(0.716)	(1.008)
	[1.61, 4.54]	[1.40, 5.68]
Male	0.927	
	(0.470)	
	[0.34, 2.50]	
Age < 60 years	0.843	
	(0.464)	
	[0.28, 2.47]	
Male (age < 60 years)		0.529 *
		(0.203)
		[0.25, 1.12]
Constant	0.056	0.055 ***
	(0.009)	(0.007)
	[0.04, 0.08]	[0.04, 0.07]
Patients	102	102
Log-likelihood	-36.73	-36.38
Chi-square test	11.73 **	12.43 **
P-value	0.039	0.014
Pseudo R-squared	0.138	0.146
Counted R-squared	0.833	0.843
AUC	0.771	0.772

## Discussion

BioGlue® is employed in various surgical procedures, particularly cranial surgeries, because of its rapid and strong adhesive qualities, which create an impermeable barrier to prevent CSF leakage. Furthermore, owing to its simplicity of use and swift application, it has the potential to conserve precious surgical time and reduce the likelihood of complications linked with extended surgical procedures. It is purported to be biocompatible, indicating that it is well-tolerated by the body without causing notable unfavorable responses or tissue harm. This is critical in neurosurgery, as the adhesive must not induce further harm to the fragile and responsive organ. It is noteworthy that although BioGlue® has demonstrated its utility in mitigating CSF leakage, several instances of its use have been reported to have unfavorable impacts in various surgical procedures.

In this study, we found that the application of BioGlue® might be related to wound reactions, especially wound problems at the surgical site resulting from a delayed chronic inflammatory process or sterile cyst confirmed by culture and histopathological examination. The presence of multinucleated giant cells without any microorganism evidence found in all cases with surgical wound problems indicated an abnormal immune response that may be associated with the BioGlue® application. Most patients commonly presented with the issue of localized edema at the site of the incision despite the satisfactory healing of their initial wound. Subsequently, the scars began to exhibit exudate emanating from specific areas. Over time, they experienced dehiscence, leading to a notable exudate being expelled. Traditional wound care initially showed signs of improvement. As time progressed, the wounds displayed greater dehiscence, requiring additional intensive intervention for drainage, including extensive I&D, along with complete evacuation of all BioGlue® and titanium plates. The purulent-like discharges at the time of wound drainage were sterile in most cases, which concords with the findings described by Pasic et al. [7]. This approach resulted in the wound issues being resolved spontaneously without antibiotic intervention. 

There were no significant differences between wound complications experienced by patients undergoing different operation methods (craniectomy or craniotomy) or different volumes of prefilled BioGlue®. Bivariate analysis using the Pearson Chi-square test and Fisher’s exact test revealed that the percentage of patients experiencing wound complications in the group that received titanium plates was statistically significantly different from those that did not receive titanium plates. In addition, no significant differences were found between the proportions of wound complications experienced by patients of different genders or age groups.

In patients with wound problems after BioGlue® application, we discovered three potential factors that may be associated with the presence of surgical wound inflammation: the utilization of titanium, the closure of skull defects, and the size of the pre-filled BioGlue® device used. We also included the gender and age of patients as control variables in the analysis, and we applied multivariate analysis using a logistic regression model to determine factors influencing wound complications. The model was estimated using the maximum likelihood estimation method. Patients treated with a 5 mL pre-filled BioGlue® device were more likely to encounter post-operative wound complications than individuals treated with the 2 mL version with odds of up to 2.8 times greater or 8.2% increased chance. Titanium plates increase the risk of surgical wound issues, whereas the use of autologous cranial bone to repair the cranial defect which acts as a barrier between the BioGlue®and the surrounding soft tissue or titanium plate decreases the likelihood of complications as seen in the lower prevalence of wound complications in the craniotomy group. These observations from the statistical analyses are consistent with clinical experience and subjective assessment. Another observation revealed that males under 60 had a slightly reduced likelihood (2.4%) of experiencing wound-related issues, which may be attributed to lesser or more well-controlled underlying diseases as compared to older patients. 

Also, from this study, the precise mechanism of wound response following the application of BioGlue® could not be definitively determined. As said in the leaflet, this incidence may relate to the tissue necrosis resulting from BioGlue®. However, there have been some literature reports that hypothesize potential mechanisms for this reaction. Luthra et al. believe the underlying cause of the adverse reactions to BioGlue® may be attributed to primary hypersensitivity and allergy to its components, particularly glutaraldehyde, or an immune response exhibited by the patients [8]. Research conducted by Slezak et al. reported that BioGlue® functioned as a foreign object, which subsequently triggered the infiltration of eosinophilic cells along with lymphocytes, plasma cells, and B cells, ultimately resulting in the development of granulomatous inflammation [9]. According to the research conducted by Fürst and Banerjee, it has been determined that BioGlue® has the potential to elicit cytotoxic on living tissues [10].

According to the WHO Drug Information Journal, Health Canada has received 13 reports of adverse incidents suspected of being associated with BioGlue®. These events were consistent with ongoing inflammatory processes upon reoperation at sites where BioGlue® had been used months earlier. BioGlue® was suspected of contributing to a sterile discharge or persistent infection. Foreign-body reactions were also reported which required the removal of BioGlue®-containing masses at the surgical site. There were cases with severe, active inflammation surrounding a BioGlue® remnant with multiple granulocytes and histiocytes and massive foreign-body reactions with numerous multinucleated giant cells [11]. In one case, a persistent concretion of BioGlue® was identified two years after application [12]. The study by Furst W and Banerjee A. warned that even though BioGlue® appears to yield clear benefits, it should be used sparingly, and its toxic potential should be borne in mind [10].

A comprehensive literature analysis revealed that multiple studies documented inflammatory reactions accompanied by localized tissue damage after the administration of BioGlue®. Economopoulos et al. hypothesized that BioGlue® could inhibit vascular growth and facilitate the formation of pseudoaneurysms when applied near vascular anastomoses [13]. Their perspective aligned with that of Baciewicz, who noticed that BioGlue® may compromise vascular anastomoses during cardiothoracic surgery [14]. This finding was also confirmed by a study by Kobayashi et al., which found inflammatory reaction-induced aortic wall weakness following elastic fiber destruction in the media of great vessels and hypothesized that BioGlue® had induced an allergic reaction in patients [15]. Rasul et al. documented two cases of spinal surgery where the application of BioGlue® resulted in a localized reaction that triggered granuloma formation, subsequently causing spinal cord compression. Following decompression surgery and the excision of BioGlue®, the pathology report identified a foreign body granuloma characterized by local inflammation, as demonstrated by the presence of foamy macrophages, lymphocytes, epithelioid cells, multinucleated giant cells, and rare neutrophils [16]. They could not identify the exact pathophysiology of such an event. They cited a study by Abuzayed et al. indicating that an increased inflammatory response may result from local immunological reactivity and tissue damage following the application of BioGlue®. This “Glue-oma” effect may occur because of polymerization and water absorption in the water sealant [17], but they could not pinpoint the exact cause-effect mechanism. They suggested that fibrosis and an over-sensitive immunological terrain could cause the formation of a granuloma and recommended applying only a thin layer as an adjunct to dural closure. Among the numerous works in the literature regarding the increased incidence of local inflammation and wound breakdown after the use of BioGlue®, one interesting paper by Hannan et al. proclaimed the safety of BioGlue® for application in extradural procedures, reporting no significant sinonasal morbidity after endoscopic transnasal transsphenoidal surgery [18].

Additional observations from this study revealed two medical variables that contributed to the delayed onset of chronic inflammation at the surgical incision site: firstly, the proximity or direct contact of BioGlue® with the adjacent subcutaneous or fat tissues or the titanium plate above, especially when cranial closure is not performed (craniectomy); and secondly, the quantity of BioGlue® utilized during the surgical procedure. Regarding the latter, we observed a higher incidence of wound reactions when the volume of pre-filled BioGlue® administered was 5 mL. Nonetheless, we cannot ascertain or establish whether the titanium plate is the causative factor or has interacted with BioGlue®, resulting in this problem. The titanium plate may or may not be the reason; nevertheless, if local inflammation develops and the plate remains in situ, it could impede the healing process or impede complete wound healing. Before drawing conclusions, further randomized controlled trials (RCTs) with larger sample sizes are needed to determine the true cause of this complication - whether it is due to titanium, BioGlue®, or a combination of both.

Limitations of the study

The limitations of this study included its retrospective design, which restricted control over confounding factors, and its inability to establish causality. Although we cannot definitively link the inflammatory tissue reaction to BioGlue®, further investigations are warranted to identify compelling evidence regarding pertinent factors, including the specific relationship between adverse events and dosage-dependent effects, the surgical technique employed, and the site of application. Another area for improvement is the sample size, which may need to be larger to enable definitive conclusions. As this is a single-center study with a limited sample size, the generalizability of results may be restricted. A multicenter study with a prospective RCT or multivariate analysis is strongly recommended and may help draw further conclusions, especially regarding cause-effect relationships.

## Conclusions

Although BioGlue® is an excellent sealant for preventing CSF leakage, neurosurgeons must consider the possibility of a delayed chronic inflammatory response in surgical wounds associated with its usage, especially when it is utilized without skull covering or comes into direct contact with subcutaneous tissue or titanium material after brain surgery. Using BioGlue® with minimal volume application is advisable to prevent delayed sterile cystic formation. The excess material of BioGlue® should be trimmed away as much as possible. In the event of a sterile cystic formation, it is advisable to perform the complete removal of BioGlue® during the initial attempt at I&D.
